# The phenotypic heterogeneity of tumor-derived cells in the bloodstream of pancreatic cancer patients

**DOI:** 10.3389/fcell.2026.1881239

**Published:** 2026-09-14

**Authors:** Chrysoula Tagari, Karolina Mangani, Michael Gogolides, Argyro Roumeliotou, Panagiota Golegou, Anastasia Xagara, Filippos Koinis, Teresa Frisan, Ioannis S. Pateras, Sophia N. Karagiannis, Athanasios Kotsakis, Galatea Kallergi

**Affiliations:** 1 Laboratory of Biochemistry and Metastatic Signaling, Division of Genetics, Cell and Developmental Biology, Department of Biology, University of Patras, Patras, Greece; 2 Department of Medical Oncology, General University Hospital of Larissa, Larissa, Greece; 3 Department of Molecular Biology and Umeå Centre for Microbial Research (UCMR), Umeå University, Umeå, Sweden; 4 Second Department of Pathology, “Attikon” University Hospital, Medical School, National and Kapodistrian University of Athens, Athens, Greece; 5 St John’s Institute of Dermatology, School of Basic and Medical Biosciences, & KHP Centre for Translational Medicine, Guy’s Hospital, King’s College London, London, United Kingdom; 6 Breast Cancer Now Research Unit, School of Cancer & Pharmaceutical Sciences, King’s College London, Innovation Hub, Guy’s Hospital, London, United Kingdom

**Keywords:** pancreatic cancer (PC), circulating tumor cells (CTCs), PD-L1, CTLA-4, STIM1, ORAI1, CD98hc

## Abstract

**Introduction:**

Pancreatic cancer (PC) remains an aggressive malignancy with poor clinical outcomes, underscoring the need for novel biomarkers to monitor patients. Immune checkpoint molecules [programmed death ligand 1 (PD-L1) and cytotoxic T-lymphocyte-associated protein 4 (CTLA-4)] have been detected in CTCs, and their expression is associated with poor prognosis. Overexpression of STIM1 and ORAI1, core regulators of calcium signaling, and the amino acid transporter chaperone CD98hc, have been shown to enhance cancer cell survival and migration. This study evaluated the expression of these molecules in PC-CTCs, alongside cytokeratin (CK, tumor cell marker) and CD45 (pan leukocyte marker).

**Methods:**

CTCs were isolated from 40 patients using Ficoll density gradient centrifugation and characterized by immunofluorescence. Additionally, 15 were analyzed using the ISET platform. PBMCs from 10 healthy donors were also evaluated. Biomarker expression was assessed using the automated VyCAP platform, followed by ACCEPT software evaluation and statistical analysis of adjunct clinical data.

**Results:**

CTCs were detected in 20% of patients, whereas all displayed (CK-/CD45-) cells with tumor characteristics and nucleus size of ≥10 μm, designated as circulating tumor-associated cells (CTACs). CTACs were significantly elevated in patients compared to healthy donors, and ROC analysis demonstrated strong discriminative capacity (AUC = 0.9083). Similarly, using the ISET platform, CTCs were detected in 26.7% (4/15), whereas CTACs were detected in 93.3% (14/15). PD-L1-positive CTCs were identified in 2.5% of patients, while PD-L1 and CTLA-4 expression on CTACs was detected in 30% and 17.5% of patients, respectively. STIM1-positive and ORAI1-positive CTCs were observed in 15% and 2.5% of patients, while expression on CTACs was detected in 70% and 10% of patients. Concerning CD98hc, 32.5% of patients harbored (CD98hc+/CD45-) cells. Interestingly, metastatic patients with PD-L1-positive CTCs exhibited shorter overall survival (OS; *p* = 0.025), while the presence of ≥2 (CD98hc+/CD45-) cells among patients who progressed within 1 year was associated with shorter progression-free survival (PFS; *p* = 0.007). Conversely, the presence of STIM1-positive CTCs correlated with improved OS (*p* = 0.021).

**Discussion:**

These findings provide a comprehensive characterization of CTCs and CTACs in PC using a multiplex biomarker panel associated with calcium signaling and immune checkpoint pathways, highlighting the pronounced heterogeneity and potential clinical relevance of these circulating cell populations.

## 1 Introduction

The innovative, minimally invasive approach of liquid biopsy has greatly transformed the oncological landscape, providing real-time, clinically significant information on disease progression and pharmacodynamics (Alix-Panabières and Pantel, 2021). Circulating tumor cells (CTCs), the cornerstone of liquid biopsy, constitute a heterogeneous and rare cancer cell population disseminated in the bloodstream from primary and metastatic sites. They serve as key initiators of metastasis through epithelial-to-mesenchymal transition (EMT) (Alix-Panabières and Pantel, 2013; Pantazaka et al., 2021) while simultaneously providing strong prognostic and predictive biomarkers through their enumeration and molecular profiling (Papakonstantinou et al., 2025; Zhao et al., 2023; Lee et al., 2023; Poruk et al., 2016). This approach holds great importance in pancreatic cancer (PC), which is characterized by a highly metastatic potential and stealthy onset, presenting substantial diagnostic and disease monitoring challenges (Wang et al., 2023; Stoop et al., 2025).

However, identifying CTCs in PC patients using conventional epithelial markers can be challenging. CTCs undergoing EMT lose the expression of epithelial markers, such as EpCAM and cytokeratins (CKs) (Gorges et al., 2012; Khoja et al., 2012; Zhao et al., 2019). This phenomenon results in the detection of atypical circulating cell populations, such as (CK-/CD45-) or (EpCAM-/CD45-) cells. These cells often exhibit mesenchymal features, further complicating the characterization of CTCs and leading to an underestimation of the true tumor burden (Xu et al., 2017; Gao et al., 2016; Zhang et al., 2015). These populations may represent EMT-associated CTCs as well as non-hematopoietic tumor-associated cell types, such as circulating cancer-associated fibroblasts (cCAFs) (Ao et al., 2015; Muchlińska et al., 2023). This has led to an increasing number of studies incorporating additional markers (Freed et al., 2023; Witek et al., 2017) and morphology-based approaches (Khoja et al., 2012) that enable the identification of distinct circulating cell subpopulations, including mesenchymal and hybrid epithelial/mesenchymal phenotypes (Zhao et al., 2019). Thus, the current study expands CTC detection beyond epithelial markers, enabling a more comprehensive characterization of tumor plasticity. Therefore, these atypical (CK-/CD45-) cells were designated as circulating tumor-associated cells (CTACs) to distinguish them from conventionally defined CK-positive CTCs and to acknowledge that their precise biological identity was not directly assessed.

The survival and growth of tumor cells depend on a wide range of pathophysiological mechanisms that hijack normal cellular processes. These mechanisms operate both intrinsically, through the rewiring of metabolic and signal transduction pathways, and extrinsically, by evading or disrupting immune surveillance. Immune checkpoint molecules, such as programmed death-ligand 1 (PD-L1) and cytotoxic T-lymphocyte-associated protein 4 (CTLA-4), normally regulate the intensity and accuracy of immune responses. They are frequently upregulated in tumor cells and CTCs, holding significant prognostic value for different types of cancers (Kallergi et al., 2018; Strati et al., 2017; Vardas et al., 2023; Buchbinder and Desai, 2016).

Likewise, calcium homeostasis represents another fundamental axis in cancer biology that controls tumor cell survival. Cancer cells maintain a steady calcium uptake mainly through store-operated calcium entry (SOCE), regulated by stromal-interaction molecule 1 (STIM1), and calcium release-activated channel protein 1 (ORAI1) (Fang et al., 2025; Ren and Li, 2023). Previous findings from our group revealed that the epigenetic factor KDM2B influences this pathway and regulates malignant progression by contributing to EMT and enhanced tumor cell migration (Zacharopoulou et al., 2018; Pantazaka et al., 2024). Notably, STIM1 and ORAI1 are highly expressed in CTCs from prostate cancer patients (Roumeliotou et al., 2024a). They have also been shown to promote tumor cell proliferation and migration in PC cells, highlighting their role in disease progression (Okeke et al., 2016; Khan et al., 2020).

To further support their increased metabolic demands, cancer cells overexpress CD98 heavy chain (SLC3A2/CD98hc), an essential chaperone for L-type amino acid transporters that also mediates β-integrin signaling, contributing to tumor cell motility and invasion (Park et al., 2024). CD98hc plays a crucial role in supporting tumor cell nutrient uptake, promoting tumor growth through the rapamycin (mTOR) signaling pathway, and enhancing antioxidant capacity by glutathione synthesis (Bianconi et al., 2022; Park et al., 2024). Although a direct mechanistic link between STIM1-ORAI1 SOCE and SLC3A2/CD98hc has not yet been fully established, STIM1-mediated SOCE has been shown to regulate redox-relevant pathways in cancer cells, including the upregulation of SLC7A11 and subsequent glutathione synthesis, which are functionally related to SLC3A2 activity in maintaining cellular oxidative balance and ferroptosis resistance (Ren et al., 2024). Thus, CD98hc limits ferroptosis, modulating the balance between tumor cell survival and death (Bianconi et al., 2022).

The aggressive nature of PC, coupled with the limitations of existing biomarkers, underscores the need for comprehensive strategies to assess real tumor burden. Therefore, this study aimed to characterize the heterogeneity of CTC and CTAC populations in PC patients, based on immune checkpoint molecules and calcium signaling-related proteins, and to explore the potential clinical relevance of these biomarkers for disease monitoring.

## 2 Materials and methods

### 2.1 Patients and healthy donor (HD) sample processing

A total of 40 treatment-naïve PC patients and 10 HDs (serving as a control group) were recruited in this study. All participants provided their written informed consent to participate in the research. Demographic and clinical characteristics of the patients are summarized in Table 1. Peripheral blood samples (20 mL) were collected from each patient and HD via venipuncture into EDTA-coated tubes, discarding the first 5 mL to prevent contamination by skin epithelial cells. PBMCs were isolated using Ficoll-Hypaque (PAN-Biotech, Aidenbach, Germany) (1.077 g/mL) density gradient centrifugation at 1800 rpm for 30 min without brakes. Under these conditions, patients’ CTCs are co-isolated within the PBMCs’ ring. PBMCs were washed 3 times with phosphate-buffered saline (PBS) and centrifuged at 1500 rpm for 10 min. Cytospins were prepared with centrifugation (2000 rpm for 2 min) of 500,000 cells onto superfrost slides (Thermo Fisher Scientific, Waltham, MA, United States). After air-drying, slides were stored at −80 °C until subsequent staining.

**TABLE 1:** Summary of pancreatic cancer (PC) patients’ clinicopathological characteristics.

Characteristics		Total PC patients (n = 40)	
		N	Percentage (%)
Age (years)	Mean ± standard deviation (SD) = 66.2 ± 11.3 Median (range) = 68 (44–86)		
Gender	Male	19	48
	Female	21	53
Histological type	Pancreatic ductal adenocarcinoma (PDAC)	31	78
	Squamous	4	10
	Undifferentiated carcinoma	1	3
	Unknown	4	10
Metastatic status	Metastatic disease	21	53
	Absence of metastatic disease	17	43
	Status unknown	2	5
Survival status	Alive	15	38
	Dead	25	63

*Percentages are rounded to the nearest whole number. Totals may not add to 100% due to this rounding.

Additionally, peripheral blood samples (10 ml) were collected from 15 patients for CTC isolation using the ISET microfiltration platform (Rarecells, Paris, France), according to the manufacturer’s instructions. Blood samples were diluted (1:10) in ISET buffer and incubated for 10 min at room temperature (RT) prior to filtration through the ISET membrane. After filtration, the membranes were air-dried and stored at −20 °C until further immunofluorescence analysis.

### 2.2 Preparation of control samples

The MIA PaCa-2 and MDA-MB-231 cell lines, derived from a patient with pancreatic adenocarcinoma and a metastatic triple-negative breast cancer (TNBC) patient, were obtained from ATCC (American Type Culture Collection, United States) and used as a control in experiments evaluating the expression of the examined biomarkers. MDA-MB-231 cells were used as an experimental positive control due to their well-characterized expression of the investigated biomarkers, while MIA PaCa-2 cells were included as a disease-relevant PC model. Expression of the investigated biomarkers in the aforementioned cell lines was further corroborated by publicly available transcriptomic datasets, including The Human Protein Atlas (HPA) (https://www.proteinatlas.org/). Cells were cultured in Dulbecco’s modified Eagle medium (DMEM, Thermo Fisher Scientific, Waltham) supplemented with 10% fetal bovine serum (FBS; PAN-Biotech), 50 U/mL penicillin, and 50 μg/ml streptomycin. Cultures were maintained at 37 °C in a humidified atmosphere with 5% CO_2_, and subcultures were performed using 0.25% trypsin-EDTA (Thermo Fisher Scientific). During the logarithmic growth phase, cancer cells were spiked into PBMCs from healthy donors at a ratio of 1000:100000 to simulate CTC conditions in the blood circulation. These spiked samples were used as positive and negative controls in the following immunofluorescence experiments.

### 2.3 Double & triple immunofluorescence stainings

Double and triple immunofluorescence stainings were performed on cytospins and ISET membrane samples, using specific antibodies targeting: CK, CD45, CTLA-4, PD-L1, STIM1, ORAI1, and CD98hc. All immunofluorescence stainings included one negative control per primary antibody and positive controls for all the examined molecules. Negative controls were prepared by omitting the respective primary antibody. This approach allowed the evaluation of immunofluorescence sensitivity and specificity, minimizing potential cross-reactivity among antibodies. Given the low frequency of CK expression in PC patients, CD98hc staining was performed in combination with CD45 without the addition of CK antibodies to evaluate the potential of CD98hc as an alternative biomarker in a broader circulating tumor-associated cell subpopulation. Representative immunofluorescence images of the positive and negative control slides are shown in Supplementary Figures S1 and S2.

In every staining experiment, samples were initially incubated with PBS for 5 min and subsequently fixed and permeabilized using ice-cold acetone/methanol (9:1, v/v) for 15 min at RT, while ISET membranes were permeabilized with 0.5% Triton X-100 for 10 min at RT. Non-specific binding was blocked by incubation with 5% FBS in PBS overnight at 4 °C. Afterwards, cells were incubated for 1 h with primary antibodies, and for 45 min with secondary antibodies.

Triple immunofluorescence stainings were performed with the following combination of antibodies: (CK/CD45/PD-L1), (CK/CD45/CTLA-4) (CK/CD45/STIM1), (CK/CD45/ORAI1). Cytokeratins (CK 8/18/19) were stained with A45 mouse antibody (Amgen Research, Munich, Germany), paired with the appropriate Alexa Fluor-conjugated anti-mouse secondary antibody (Invitrogen, Waltham, MA, United States; A31570 or A11017). CD45 was detected either with CD45-Alexa 647 conjugated antibody (Santa Cruz Biotechnology, Dallas, TX, United States; sc-1178 AF647) or with CD45 rabbit (D9M8I) (Cell Signaling Technology, Danvers, MA, United States; #13917) and Alexa 488 anti-rabbit antibody (Invitrogen A21206), depending on the combination of the antibodies. PD-L1 was stained using PD-L1 goat (Novus Biologicals, Littleton, CO, United States; NB-300–903) and Alexa 488 anti-goat antibody (Invitrogen A21467). CTLA-4 detection was performed with CTLA-4-Alexa 488 conjugated antibody (sc-376016 AF488). STIM1 was targeted with Stim1-Alexa 546 conjugated antibody (sc-166840 AF546), and ORAI1 was detected with ORAI1-Alexa 647 conjugated antibody (sc-377281 AF647).

CD98hc was detected by double immunofluorescence staining experiments (CD98hc/CD45). Particularly, CD98hc was detected with SF25 IgE antibody (produced and kindly provided by Prof. Karagiannis’ lab, King’s College London), followed by APC anti-IgE (633) antibody (BioLegend, San Diego, CA, United States; Cat. 325508) (Pellizzari et al., 2021). The (STIM1/CD45) staining was specifically performed on cytospin slides of HD-derived PBMCs, while the ISET membranes were stained with (CK/CD45) antibodies for CTC characterization.

Finally, all samples were mounted with ProLong Antifade medium containing DAPI (Cell Signaling Technology) for nuclear staining and stored at −20 °C until further analysis.

### 2.4 Sample analysis

CTC identification was based on expression of the epithelial marker CK and the absence of the leukocyte marker CD45. Additional cytomorphological criteria commonly applied for CTCs were also used, including cell diameter ≥10 μm, high nuclear-to-cytoplasmic ratio, and irregular nuclear morphology (Khoja et al., 2012; Bobek et al., 2014; Song et al., 2021). Using these combined criteria, a distinct subpopulation of atypical (CK-/CD45-) cells with malignant features (cell diameter ≥10 μm) was also identified and categorized as CTACs. Since this cell population lacks CK expression, it may include tumor-derived cells that have undergone phenotypic changes associated with EMT. However, as their malignant nature was not molecularly validated in the present study, they were designated as CTACs rather than CTCs. The same morphological criteria were applied to samples from HDs, establishing a threshold (based on the highest number of these cells in HDs), thereby minimizing the risk of false positive results in cancer patients.

Sample analysis was performed following a two-step approach. Slides were first analyzed using the VyCAP automated imaging system (VyCAP B.V., Enschede, Netherlands). Images from patient and control samples were acquired using predefined exposure settings established from positive and negative controls. Subsequently, the produced frames were analyzed with the open-source ACCEPT software (v1.1, University of Twente, Enschede, Netherlands; https://github.com/LeonieZ/ACCEPT), to validate findings and identify CTACs (Zeune et al., 2017). The ACCEPT gating thresholds were determined using numerous manually identified patient CTCs and CTACs. These cells were analyzed in ACCEPT, and their morphological and fluorescence parameter distributions were compared with those of PBMCs. The final thresholds were selected to optimally distinguish tumor-associated cell populations from leukocytes and were subsequently applied uniformly across all patient and HD samples.

The following two gates were applied to every sample to detect CTCs and CΤΑCs:

- 1st PC gate:

CD45 → Mean Intensity ≤0

DAPI → Size >400

DAPI → Eccentricity >0.2 & ≤ 0.8

DAPI → Perimeter >100 & ≤185

DAPI → Perimeter-to-area ratio >0.95 & ≤ 1.15

- 2nd PC gate:

CD45 → Overlay with DNA >0.39 & ≤ 0.5

DAPI → Size >400

DAPI → Eccentricity >0.9

Final identification of CTCs and CTACs was performed in a blinded manner with respect to clinical data.

### 2.5 Statistical analysis

Statistical analyses to explore the potential clinical and prognostic significance of the identified phenotypes were carried out using IBM SPSS Statistics software (version 29.0; IBM Corp., Armonk, NY, United States) and GraphPad Prism software (version 8.0.1; GraphPad Software, San Diego, CA, United States), with a threshold of statistical significance set at *p* < 0.05. Progression-free survival (PFS) was defined as the period, calculated in months, from enrollment in this study to the date of relapse, death, or the last follow-up, whichever occurred first. Overall survival (OS) was defined as the duration, calculated in months, from enrollment in this study to the date of death from any cause or last follow-up. Survival analyses (Kaplan-Meier analysis, Cox regression analysis) were performed to explore any associations between the presence of CTCs and/or CTACs and patients’ OS and PFS. Survival curves were created using RStudio software (version 2026.04.0 + 526, Posit Software, Boston, MA, United States). Spearman’s rank correlation coefficient was employed to examine correlations between different tumor-derived cell phenotypes across immunofluorescent stainings. McNemar’s test was used to evaluate differences in the number of patients exhibiting distinct phenotypes.

The Kolmogorov-Smirnov test was used to assess the data’s normal distribution. As the data were not normally distributed, non-parametric tests were applied. Paired comparisons between the mean counts of CTCs and CTACs per patient were carried out with the Wilcoxon signed-rank non-parametric test. The Mann-Whitney U test was used to compare CTAC cell counts between PC patients and HDs, while receiver operating characteristic (ROC) curve analysis was performed to evaluate their discriminatory ability between the two groups.

## 3 Results

### 3.1 Phenotypic plasticity of tumor-derived circulating cells in PC patients

Among all patients, two distinct tumor-derived cellular subtypes were detected: typical CTCs (CK+CD45-) and atypical CTACs with a (CK-/CD45-, ≥10 μm nuclei size) phenotype. In all the staining experiments, CTCs were detected in 20% of patients, whereas all displayed (CK-/CD45-) cells with tumor characteristics and nucleus size of ≥10 μm, designated as circulating tumor-associated cells (CTACs). Mean CTAC counts per patient calculated across four independent immunofluorescent experiments in cytospins were significantly higher than those of CTCs (*p* < 0.0001), as shown in Figure 1A. Each experiment analyzed 10^6^ PBMCs. Representative images of CTCs and CTACs from both the VyCAP platform and the ACCEPT software are illustrated in Figures 1B,C, respectively. The presence of CTACs was also evaluated in samples from 10 healthy donors (Supplementary Table S1). Although these cells were also detected within this group, their number was significantly lower [median 1 (range 0–4)] (*p* < 0.0001), compared to those of PC patients [median 4.875 (range 1–41.5)] (Figure 1D), reinforcing their association with a malignant context. ROC analysis further demonstrated strong discrimination ability of CTAC counts between PC patients and HDs, yielding an AUC of 0.9038 (95% CI: 0.80–1.000, *p* < 0.0001; Figure 1E). Based on these results and the upper range observed in HDs, patients were classified as CTAC-positive when they harbored >4 CTACs in their blood for the subsequent survival and phenotypic analyses. Using this threshold, Cox regression analysis showed worse OS for CTAC-positive metastatic patients [log-rank *p* = 0.03, Hazard ratio (HR) = 1.26, (95% CI: 1.02–1.54); Table 2].

**FIGURE 1:**
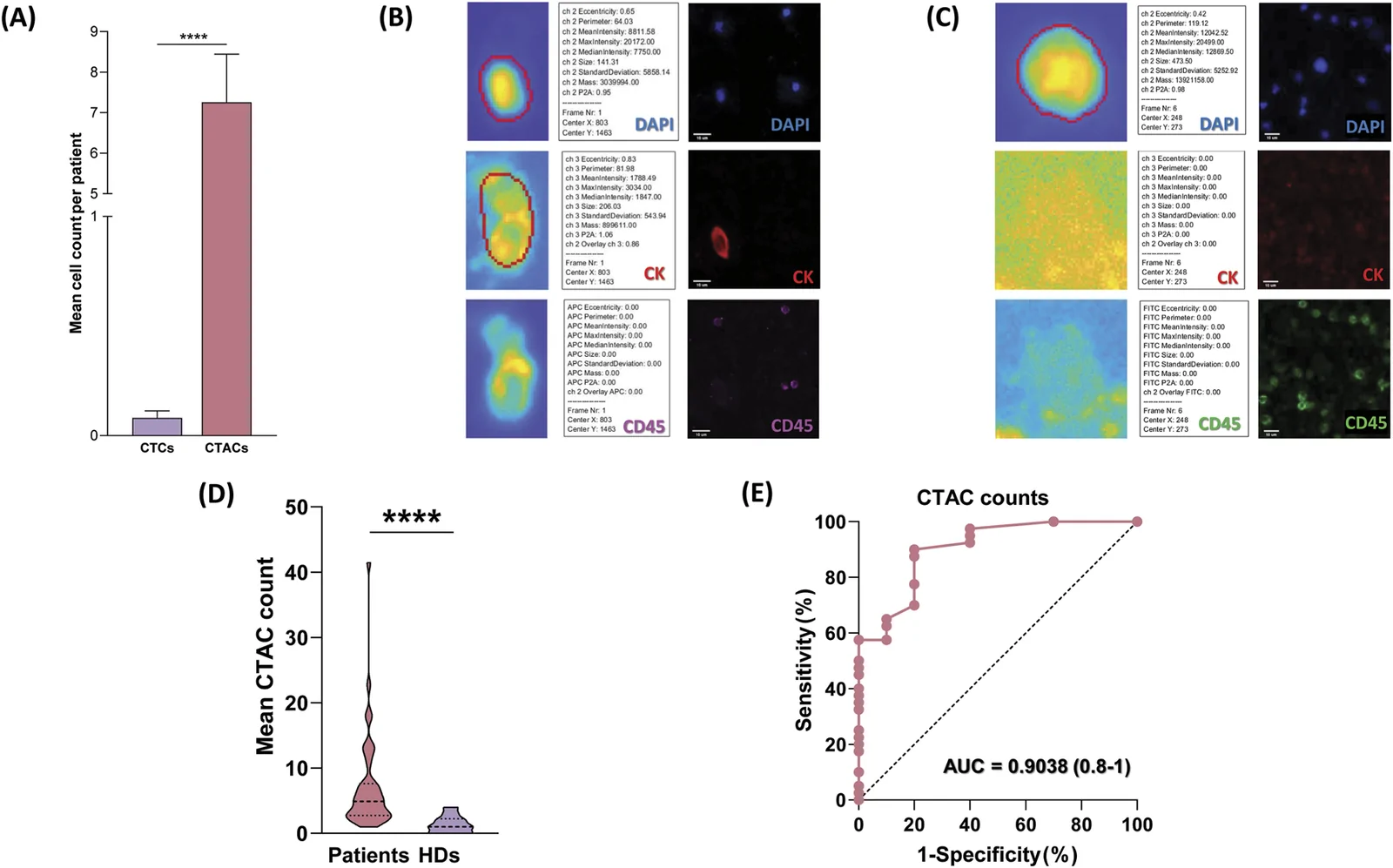
Circulating tumor cells (CTCs) and circulating tumor-associated cells (CTACs) detected in pancreatic cancer (PC) patients and the potential clinical relevance of CTACs **(A)** Mean number of CTCs and CTACs per patient across immunofluorescence staining experiments. Error bars represent the standard error of the mean (SEM). Statistical significance was determined using the Wilcoxon signed-rank test, with significance indicated as follows: **** *p* ≤ 0.0001 **(B)** Representative images of a CTC using the open source ACCEPT software. Shown are the fluorescence channels for DAPI (blue), CK (red), and CD45 (purple). Images were acquired using the VyCAP platform at 20× magnification. Scale bars represent 10 μm **(C)** Representative images of a CTAC using the open source ACCEPT software. Shown are the fluorescence channels for DAPI (blue), CK (red) and CD45 (green). Images were acquired using the VyCAP platform at 20× magnification. Scale bars represent 10 μm **(D)** Mean CTAC number detected among PC patients and healthy donors (HDs). Data are presented as violin plots with the solid line indicating the median and the dotted lines representing the interquartile range. Statistical significance was assessed using the Mann-Whitney U test and is marked as follows: **** *p* ≤ 0.0001 **(E)** Receiver Operating Characteristic (ROC) curve presenting the discriminative ability of CTAC counts to distinguish PC patients from HDs (AUC: 0.9038; *p* < 0.0001).

**TABLE 2:** Cox regression analysis of overall survival in circulating tumor-associated cell (CTAC)-positive metastatic patients.

Variable	Hazard Ratio (HR)	95% CI	*p*-value
Average CTAC cell count	1.26	1.02–1.54	0.030

Similarly, using the ISET platform, CTCs were detected in 26.7% (4/15) of patients, whereas CTACs were detected in 93.3% (14/15) of patients (Supplementary Table S2).

### 3.2 Phenotypic characterization of CTCs and CTACs in PC patients

#### 3.2.1 Immune checkpoint markers expression

Immunofluorescence staining experiments with the following combination of antibodies [CK/PD-L1/CD45] and [CK/CTLA-4/CD45] revealed a clear predominance of CTACs over CTCs. Specifically, regarding the (CK/PD-L1/CD45) staining, CTCs were detected in 8% of patients (3/40), whereas CTACs were identified in 80% of patients (32/40) (Supplementary Table S3). Representative images of PD-L1 and CTLA-4 expression in CTCs and CTACs, are shown in Figures 2A,B, respectively.

**FIGURE 2:**
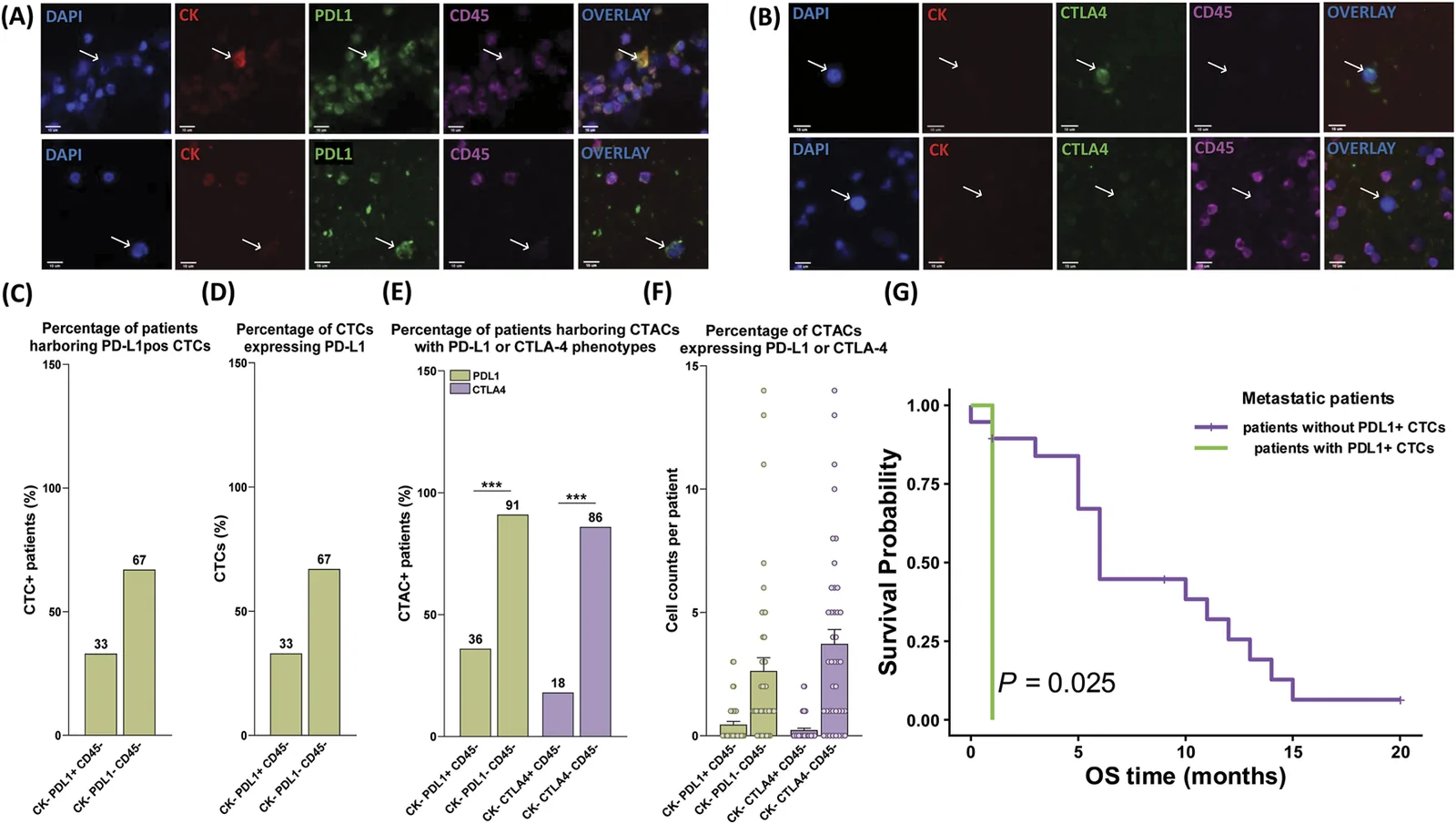
Immune checkpoint marker expression in circulating tumor cells (CTCs) and circulating tumor-associated cells (CTACs) detected in pancreatic cancer (PC) patients **(A)** Representative images of (CK+/PD-L1+/CD45-) and (CK-/PD-L1+/CD45-) cells detected, stained with CK (red), PD-L1 (green) and CD45 (purple). Nuclei were stained with DAPI (blue). Images were acquired using the VyCAP platform at 20× magnification. Scale bars represent 10 μm **(B)** Representative images of (CK-/CTLA-4+/CD45-) and (CK-/CTLA-4-/CD45-) cells detected, stained with CK (red), CTLA-4 (green) and CD45 (purple). Nuclei were stained with DAPI (blue). Images were acquired using the VyCAP platform at 20× magnification. Scale bars represent 10 μm **(C)** Percentage of patients harboring CTCs, with PD-L1 expression **(D)** Frequency of detected PD-L1 phenotypes among the total isolated CTCs **(E)** Percentage of patients harboring CTACs, with PD-L1 or CTLA-4 expression. Statistical significance was determined using McNemar’s test, with significance indicated as follows: *** *p* ≤ 0.001 **(F)** Distribution of CTAC phenotypes based on PD-L1 and CTLA-4 expression across patients. Each dot represents the number of cells of the indicated phenotype per patient. Error bars represent the standard error of the mean (SEM) **(G)** Kaplan-Meier survival curve showcasing that PC metastatic patients expressing PD-L1-positive CTCs exhibit shorter overall survival (OS: log-rank *p* = 0.025, Hazard Ratio (HR) = 9.163). Statistical significance was determined using the log-rank test.

Regarding PD-L1 expression among CK-positive patients, 33% (1/3) of them harbored cells with the (CK+/PD-L1+/CD45-) phenotype, while 67% (2/3) had cells with the (CK+/PD-L1-/CD45-) phenotype (Figure 2C). Among the total isolated CTCs, 33% (1/3) were classified as PD-L1-positive and 67% (2/3) as PD-L1-negative (Figure 2D).

In the cohort of CTAC-positive patients (>4 CTACs), 36% (8/22) harbored PD-L1-positive CTACs, while the (CK-/PD-L1-/CD45-) phenotype was present at 91% (20/22, *p* < 0.001, Figure 2E). The frequency of PD-L1-positive CTACs was 12% (13/106) compared to 88% (93/106) of the PD-L1-negative CTACs (Figure 2F).

Further analysis regarding disease status (metastatic/non-metastatic) for (CK/PD-L1/CD45) staining revealed that among the non-metastatic patients, 2 harbored CTCs, and one of them (1/2) had PD-L1-positive CTCs (Supplementary Table S4A). In contrast, in metastatic patients, only 1 patient was CTC-positive, and the detected CTC phenotype lacked PD-L1 expression (Supplementary Table S4A,B).

Considering CTACs and disease status, 20% (2/10) of the non-metastatic patients exhibited PD-L1-positive CTACs (Supplementary Table S4A). Within this cohort, 11% (4/37) of the total isolated CTACs were PD-L1-positive (Supplementary Table S4B). In metastatic patients, the respective numbers were 55% (6/11 patients) (Supplementary Table S4A) and 14% (9/63 CTACs) (Supplementary Table S4B).

Statistical comparisons among phenotypes from the (CK/PD-L1/CD45) staining in the full cohort of 40 patients are summarized in Table 3.

**TABLE 3:** Summary of significant differences among circulating tumor cell (CTC) and circulating tumor-associated cell (CTAC) phenotypes, in the (CK/PD-L1/CD45) staining.

Pairwise McNemar test comparisons	*p*-value
PD-L1+ CTCs < PD-L1+ CTACs	0.006
PD-L1- CTCs < PD-L1+ CTACs	0.003
PD-L1+ CTCs < PD-L1- CTACs	< 0.001
PD-L1- CTCs < PD-L1- CTACs	< 0.001

Concerning the (CK/CTLA-4/CD45) staining, no CTCs were detected, while the majority of patients (85%, 34/40) had detectable CTACs. Based on the HD threshold (>4) 55% of patients (22/40) were classified as CTAC-positive (Supplementary Table S5). Amongst them the (CK-/CTLA-4-/CD45-) phenotype was significantly more frequent (*p* < 0.001) at 86% (19/22), while 18% (4/22) displayed the (CK-/CTLA-4+/CD45-) phenotype (Figure 2E). Among the total analyzed CTACs, 96% (122/127) exhibited the (CK-/CTLA-4-/CD45-) phenotype and 4% (5/127) of them the (CK-/CTLA-4+/CD45-) phenotype (Figure 2F).

Analysis concerning disease status revealed that in the (CK/CTLA-4/CD45) staining, only a small subset of non-metastatic CTAC-positive patients (20%, 2/10) (Supplementary Table S6A) presented CTLA-4-positive CTACs (Supplementary Table S6A). CTLA-4 expression was detected in only 4% (2/49) of the total CTACs in this cohort (Supplementary Table S6B). Similarly, among metastatic CTAC-positive patients, the (CK-/CTLA-4+/CD45-) phenotype was detected in 18% (2/11) of patients (Supplementary Table S6A), and CTLA-4-positive CTACs accounted for only 3% (2/70) of the total identified CTACs (Supplementary Table S6B).

Statistical comparisons of the phenotypes’ frequencies for immune checkpoint stainings across patients are summarized in Table 4.

**TABLE 4:** Summary of significant differences among circulating tumor cell (CTC) and circulating tumor-associated cell (CTAC) phenotypes, in the (CK/PD-L1/CD45) and (CK/CTLA-4/CD45) stainings.

Pairwise McNemar test comparisons	*p*-value
Non-metastatic patients: PD-L1+ CTCs < PD-L1- CTACs	< 0.001
Non-metastatic patients: PD-L1- CTCs < PD-L1-CTACs	< 0.001
Metastatic patients: PD-L1+ CTCs < PD-L1- CTACs	0.001
Metastatic patients: PD-L1+ CTCs < PD-L1+ CTACs	0.016

#### 3.2.2 Clinical relevance of immune checkpoint molecule expression

Analysis of patients’ clinical data regarding PD-L1 and CTLA-4 phenotypes yielded a statistically significant reduction of OS in metastatic patients with PD-L1 expressing CTCs. Particularly, Kaplan-Meier analysis showed that metastatic patients harboring PD-L1-positive CTCs exhibited shorter OS compared to patients who did not harbor these cells [1.00 months vs. 6.00 months (95% CI: 4.97–7.027) respectively; log-rank *p* = 0.025; Figure 2G]. Cox regression analysis also revealed a similar trend (HR 9.163, 95% CI: 0.83–101.07), although this did not reach statistical significance (*p* = 0.071).

#### 3.2.3 Calcium signaling biomarker expression

The presence of CTACs was further confirmed in 95% of patients (38/40) with the (CK/STIM1/CD45) staining. Firstly, in line with the previous observations, CTCs were detected in only 15% (6/40) of cases (Supplementary Table S7). Phenotypic classification of CTCs revealed that all patients with CTCs (100%, 6/6) carried the (CK+/STIM1+/CD45-) phenotype, while 33% (2/6) harbored also the (CK+/STIM1-/CD45-) phenotype (Figure 3A). Among the total detected CTCs, 75% of them were categorized as STIM1-positive, while 25% were STIM1-negative (Figure 3B).

**FIGURE 3:**
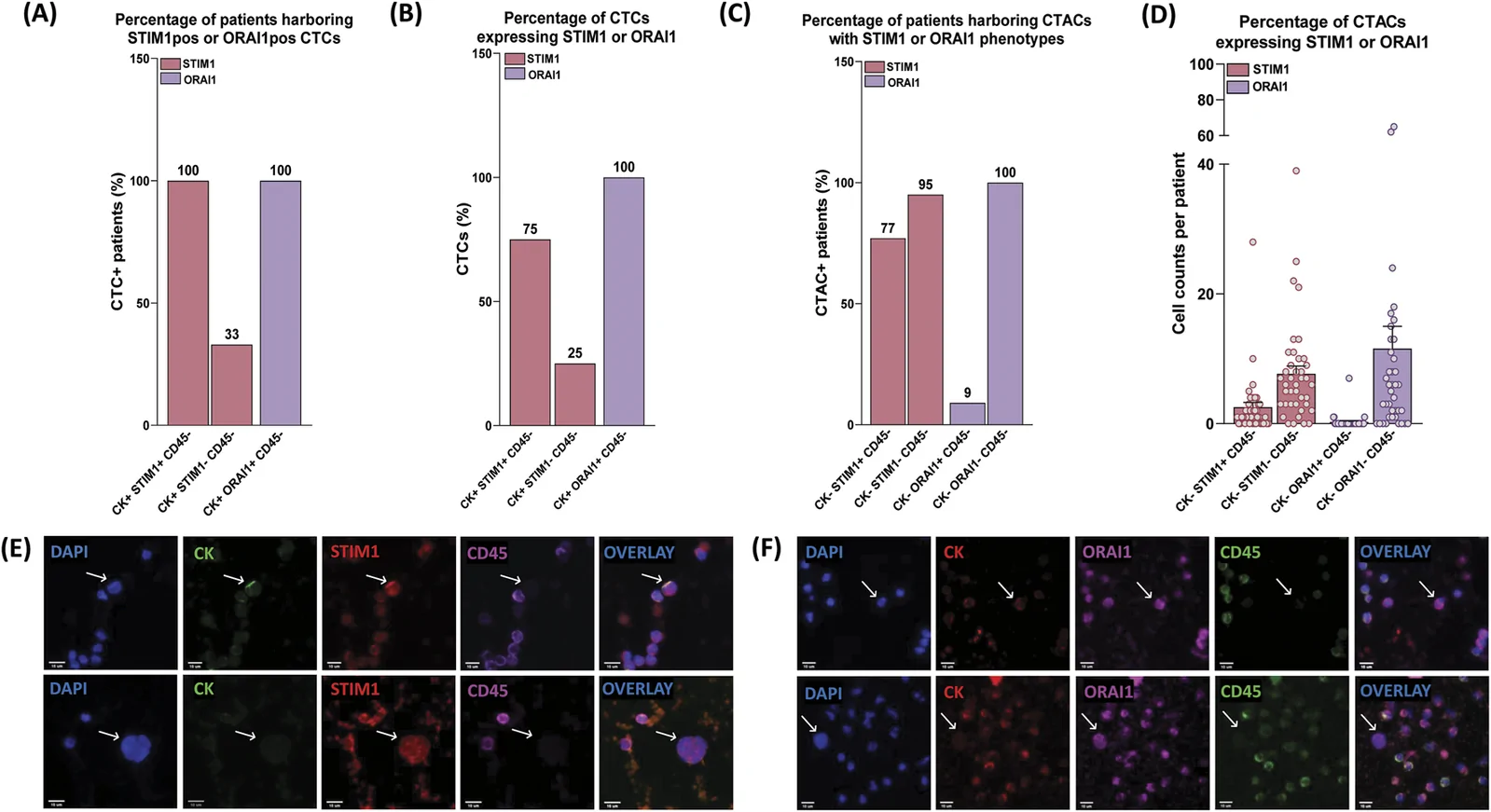
Phenotypic patterns of STIM1 and ORAI1 in circulating tumor cells (CTCs) and circulating tumor-associated cells (CTACs) detected in pancreatic cancer (PC) patients. **(A)** Percentage of patients harboring CTCs with STIM1 or ORAI1 expression. **(B)** Frequency of the detected STIM1 or ORAI1 phenotypes among the total isolated CTCs. **(C)** Percentage of patients harboring CTACs with STIM1 or ORAI1 expression. **(D)** Distribution of CTAC phenotypes based on STIM1 and ORAI1 expression across patients. Each dot represents the number of cells of the indicated phenotype per patient. Error bars represent the standard error of the mean (SEM). **(E)** Representative images of STIM1 expression in CTCs and CTACs detected, stained with CK (green), STIM1 (red), and CD45 (purple). Nuclei were stained with DAPI (blue). Images were acquired using the VyCAP platform at 20× magnification. Scale bars represent 10 μm. **(F)** Representative images of ORAI1 expression in CTCs and CTACs detected, stained with CK (red), ORAI1 (purple), and CD45 (green). Nuclei were stained with DAPI (blue). Images were acquired using the VyCAP platform at 20× magnification. Scale bars represent 10 μm.

Regarding CTACs analysis, among the CTAC-positive patient group (>4 CTACs), the (CK-/STIM1+/CD45-) phenotype was observed in 77% (17/22), while the (CK-/STIM1-/CD45-) phenotype was also present in 95% (21/22) of the patients (Figure 3C). In contrast to the expression patterns observed in CTCs, only 20% of the total isolated CTACs (64/316) displayed the (CK-/STIM1+/CD45-) phenotype, whereas 80% (252/316) exhibited the (CK-/STIM1-/CD45-) phenotype (Figure 3D).

Considering disease stage, all the CTC-positive non-metastatic patients (100%, 5/5) displayed STIM1-positive CTCs (100%, 5/5), with one patient (20%, 1/5) also presenting STIM1-negative CTCs (Supplementary Table S8A). Non-metastatic patients’ CTCs were classified as STIM1-positive at 83% (5/6) and STIM1-negative at 17% (1/6) (Supplementary Table S8B). In the cohort of metastatic patients, only one was CK-positive. (Supplementary Table S8A,B).

Regarding disease status, 90% (9/10) of the CTAC-positive non-metastatic patients were STIM1-positive, and the frequency of STIM1 expression was 14% (24/167) (Supplementary Tables S8A,B). Among metastatic patients, STIM1-positive CTACs were identified in 91% (10/11) (Supplementary Table S8A), while the detected CTACs displayed STIM1 expression at 28% (30/107, Supplementary Table S8B). Statistical comparisons between phenotypes are summarized in Table 5.

**TABLE 5:** Summary of significant differences among circulating tumor cell (CTC) and circulating tumor-associated cell (CTAC) phenotypes, in the (CK/STIM1/CD45) staining.

Pairwise McNemar test comparisons	*p*-value
STIM1+ CTCs < STIM1+ CTACs	< 0.001
STIM1+ CTCs < STIM1- CTACs	< 0.001
STIM1- CTCs < STIM1+ CTACs	< 0.001
STIM1- CTCs < STIM1- CTACs	< 0.001

Regarding (CK/ORAI1/CD45) staining, CTCs were detected in only one patient, and all of them were ORAI1-positive (Figure 3A). Conversely, CTACs were detected in the majority of patients (83%, 33/40) (Supplementary Table S9).

Among the CTAC-positive patients, all cases presented the (CK-/ORAI1-/CD45-) phenotype (100%, 22/22), while only 9% (2/22) displayed the (CK-/ORAI1+/CD45-) phenotype (Figure 3C). Only 1% (3/321) of CTACs detected were ORAI1-positive (Figure 3D).

Statistical comparisons between different phenotypes observed at the (CK/ORAI1/CD45) staining are summarized in Table 6.

**TABLE 6:** Summary of significant differences among circulating tumor cell (CTC) and circulating tumor-associated cell (CTAC) phenotypes, in the (CK/ORAI1/CD45) staining.

Pairwise McNemar test comparisons	*p*-value
ORAI1+ CTCs < ORAI1- CTACs	< 0.0001

Regarding disease stage, 2 CTCs with ORAI1-positive phenotypes were detected in one non-metastatic patient (1/10) (Supplementary Tables S10A, S10B). In contrast, no CTCs were identified in metastatic patients (Supplementary Tables S10A, S10B). Furthermore, 10% (1/10) of the non-metastatic patients harbored CTAC-positive cells (Supplementary Table S10A), with 1% ORAI1 positivity (1/88), (Supplementary Table S10B). Consistently, CTAC-positive metastatic patients harbored ORAI1-positive CTACs at 9% (1/11) (Supplementary Table S10A), while the frequency of ORAI1-positive CTACs was also 1% (1/168) (Supplementary Table S10B).

Statistical comparisons between different phenotypes in both staining panels, stratified by disease status, are summarized in Table 7.

**TABLE 7:** Summary of significant differences among circulating tumor cell (CTC) and circulating tumor-associated cell (CTAC) phenotypes in the (CK/ORAI1/CD45) and (CK/STIM1/CD45) stainings.

Pairwise McNemar test comparisons	*p*-value
Non-metastatic patients: STIM1+ CTCs < STIM1- CTACs	0.006
Non-metastatic patients: STIM1- CTCs < STIM1+ CTACs	0.021
Non-metastatic patients: STIM1- CTCs < STIM1- CTACs	< 0.001
Non-metastatic patients: ORAI1+ CTCs < ORAI1- CTACs	< 0.001
Metastatic patients: STIM1+ CTCs < STIM1+ CTACs	< 0.001
Metastatic patients: STIM1+ CTCs < STIM1- CTACs	< 0.001
Metastatic patients: STIM1- CTCs < STIM1+ CTACs	< 0.001
Metastatic patients: STIM1- CTCs < STIM1- CTACs	< 0.001

STIM1-positive CTCs were also correlated with ORAI1-positive CTCs (rho = 0.348, *p* = 0.028) as well as ORAI1-positive CTACs (rho = 0.504, *p* < 0.001; Supplementary Table S11). Representative images of STIM1 and ORAI1 expression in CTCs and CTACs are shown in Figures 3E,F.

#### 3.2.4 CD98hc expression

Considering the low CK expression observed in PC patients’ CTCs, the current study used CD98hc as a potential biomarker in combination with morphological criteria and the exclusion marker CD45, aiming to identify a substantial number of cells for further clinical validation. Therefore, the samples were characterized by the following combination of antibodies: (CD98hc/CD45). It is important to note that since CK was not included in this panel, it was not possible to distinguish CTCs from CTACs, and the (CD45-) population may include both cell types (Supplementary Table S12). Representative images of CD98hc expression in CD45-negative ≥10 μm cells are shown in Figures 4A,B.

**FIGURE 4:**
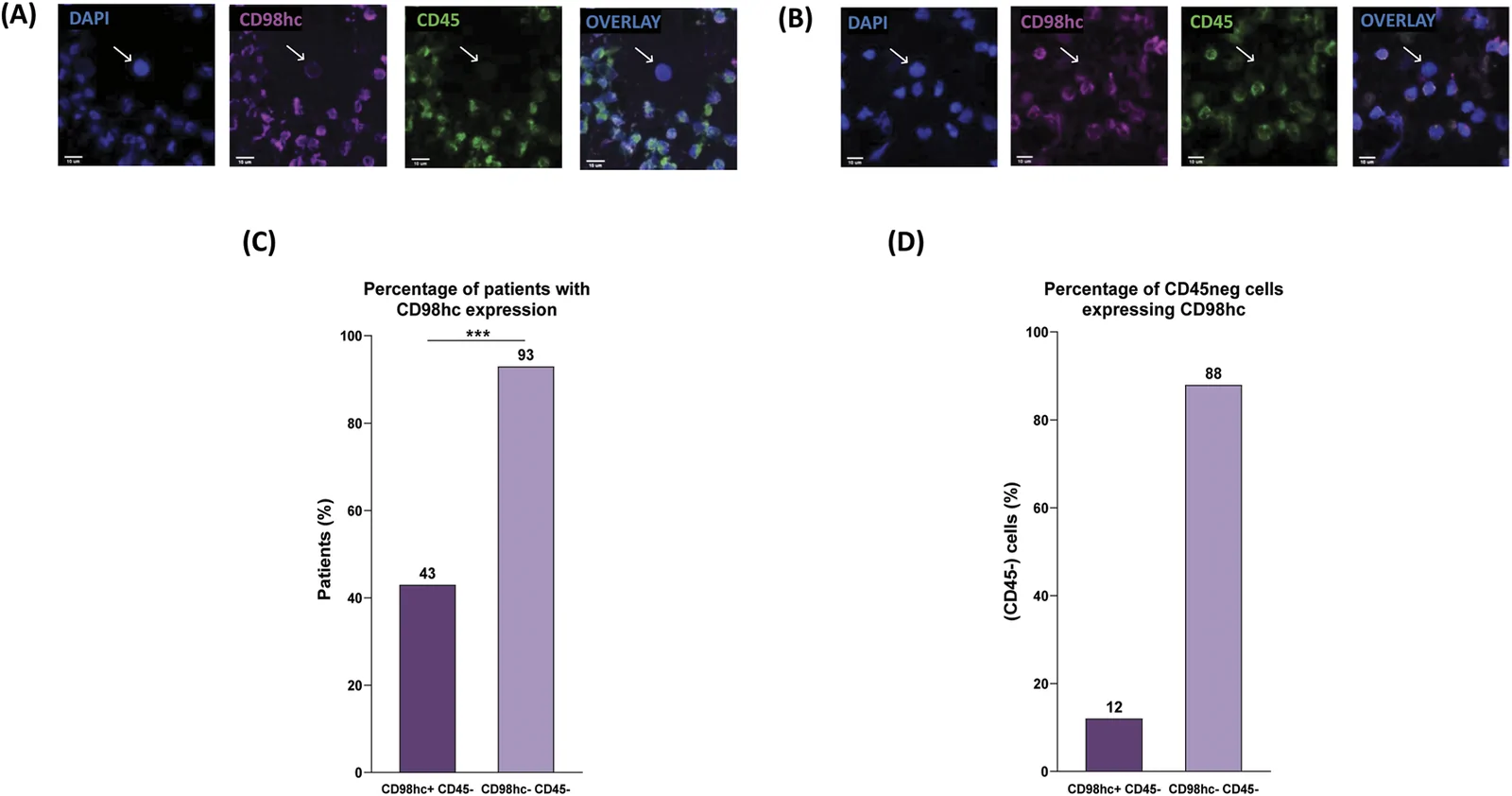
CD98hc expression in (CD45-) cells detected in pancreatic cancer (PC) patients. Representative images of **(A)** (CD98hc+/CD45-) cell identified and **(B)** (CD98hc-/CD45-) cell identified, stained with CD98hc (purple) and CD45 (green) antibodies. Nuclei were stained with DAPI (blue). Images were acquired using the VyCAP platform at 20× magnification. Scale bars represent 10 μm **(C)** Percentage of patients harboring (CD45-) ≥10 μm cells with CD98hc expression. Statistical significance was determined using the McNemar’s test, with significance indicated as follows: *** *p* ≤ 0.001 **(D)** Mean percentages of total (CD45-) ≥10 μm cells expressing CD98hc.

Among the total patients, 43% (13/30) exhibited (CD98hc+/CD45-) cells, whereas (CD98hc-/CD45-) cells were significantly more frequent [93% (28/30), *p* < 0.0001] (Figure 4C). Moreover, among the total (CD45-) cells detected, the (CD98hc-/CD45-) phenotype was observed at 88%, followed by the (CD98hc+/CD45-) phenotype at 12% (Figure 4D).

Assessment of CD98hc expression regarding the disease status of the patients revealed that non-metastatic patients harbored (CD98hc+/CD45-) cells at 50% (6/12) (Supplementary Table S13A). Non-metastatic patients exhibited CD98hc expression at 17% (6/83) (Supplementary Table S13B). Similarly, metastatic patients harbored (CD98hc+/CD45-) cells at 35% (6/17) (Supplementary Table S13A). Among the total (CD45-) cells observed in this cohort, the frequency of the (CD98hc+/CD45-) phenotype was at 10% (10/104), Supplementary Table S13B).

#### 3.2.5 Clinical relevance of calcium signaling-related molecules

Evaluation of the clinical relevance of STIM1 and ORAI1 expression patterns interestingly, revealed that the presence of STIM1-positive CTCs was correlated with better OS compared to patients who did not exhibit these cells [15 months vs. 6 months (95% CI: 1.02–10.98), respectively; log-rank *p* = 0.021; Figure 5A]. Cox regression analysis also revealed a similar trend (HR = 0.164, 95% CI: 0.019–1.088), although it did not reach statistical significance (log-rank *p* = 0.06).

**FIGURE 5:**
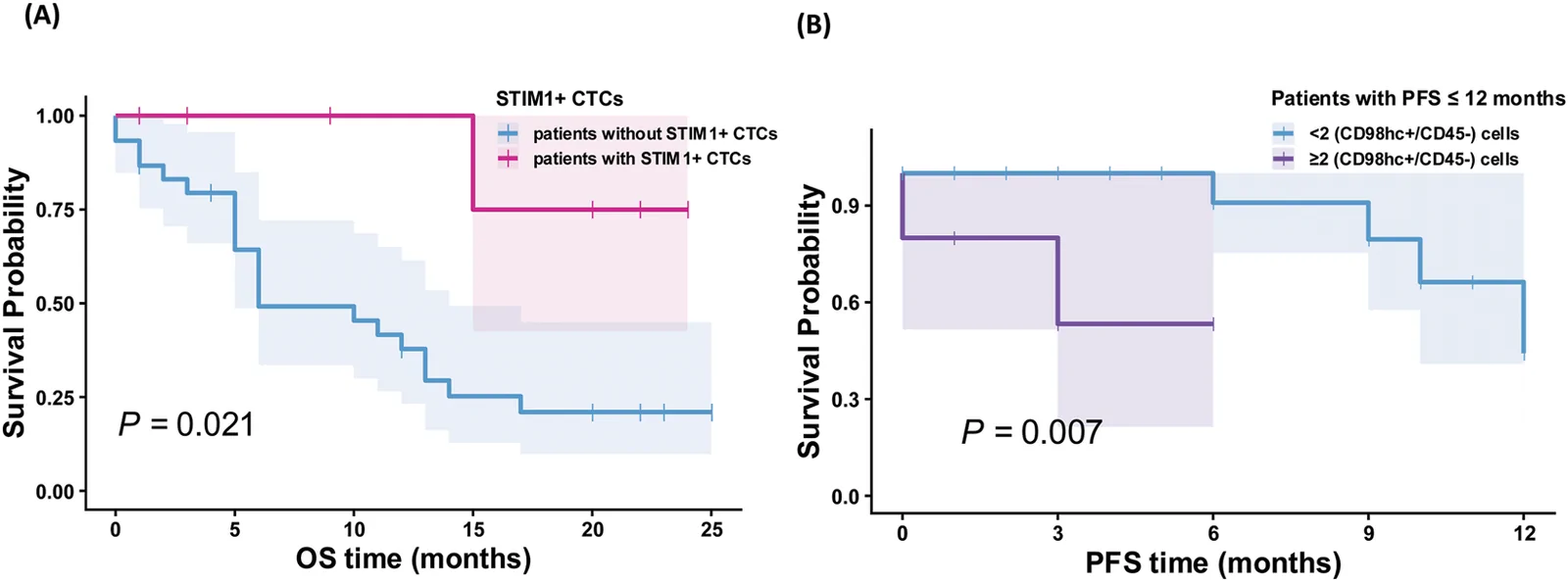
Clinical significance of calcium signaling-related molecules. Kaplan-Meier survival curves showing that: **(A)** Pancreatic cancer (PC) patients with STIM1-positive circulating tumor cells (CTCs) correlated with better overall survival (OS: log-rank *p* = 0.021, Hazard Ratio (HR) = 0.164) **(B)** Among patients who progressed within 1 year, the presence of ≥2 (CD98hc+/CD45-) cells was associated with shorter progression-free survival (PFS: log-rank *p* = 0.007, HR = 13.6). Statistical significance was determined using the log-rank test.

Furthermore, CD98hc expression was also associated with patients’ clinical data. Survival analysis revealed that, within the subgroup of patients who experienced disease progression in less than one-year (PFS ≤12 months), the presence of 2 or more (CD98hc+/CD45-) cells was associated with shorter PFS compared to patients who did not exhibit this phenotype [3 months vs. 12 months (95% CI: 8.26–15.74), respectively; log-rank *p* = 0.007); Figure 5B]. Cox regression analysis within this group also confirmed a significant difference in PFS [log-rank *p* = 0.036, HR = 13.6, (95% CI: 1.19–156.41)].

## 4 Discussion

PC is usually a very aggressive subtype, advancing asymptomatically in the initial stages. This leads to late diagnosis, when the disease has already spread to distant sites (Wang et al., 2023; Stoop et al., 2025). Since monitoring the disease with repeated tissue biopsies is physically challenging and often impossible for patients, liquid biopsy and particularly CTCs have emerged as promising real-time tools to address this issue (Lianidou and Pantel, 2019).

However, effective detection of CTCs in PC patients is puzzling due to their significant heterogeneity. This heterogeneity is attributed to EMT, a hallmark of metastasis. As a result, in addition to epithelial CTCs, distinct subpopulations with hybrid epithelial/mesenchymal characteristics or fully mesenchymal phenotypes are also detected in the bloodstream of these patients and are clinically relevant, being associated with advanced stage, distant metastasis, and shorter recurrence-free survival (Zhao et al., 2019; Semaan et al., 2021; Zhao et al., 2021).

PC heterogeneity is also reflected in CTC physiology. Studies relying on epithelial epitope-dependent approaches, such as CellSearch, have reported low and controversial CTC recovery rates ranging from approximately 7%–22% in some PC cohorts (Hugenschmidt et al., 2021; Bidard et al., 2013), whereas size-based isolation methods have achieved higher rates (up to 89%) (Khoja et al., 2012; Lee et al., 2019). Such findings highlight that, depending only on epithelial markers, may overlook certain CTC subpopulations and underestimate tumor burden in PC. For this reason, this study combined morphological criteria with epithelial markers to detect tumor-associated subpopulations, including CK-negative cells.

In the current study, CTACs were detected at higher percentages compared to CTCs in PC patients across all staining experiments through Ficoll-based isolation and size-based filtration with the ISET platform, with the CTC recovery rates also appearing comparable between the two methods (Figure 1A; Supplementary Table S2). These cells were significantly enriched in patients compared to HDs (median counts of 4 vs. 1, respectively). ROC analysis further indicated strong discriminatory ability between patients and HDs (Figures 1D,E). Additionally, CTACs showed potential clinical relevance as higher counts among metastatic patients significantly correlated with worse OS [Cox regression, log-rank *p* = 0.03, HR = 1.26 (95% CI: 1.02–1.54)]. These findings are consistent with previous studies showing that epithelial markers such as EpCAM, CK, and E-cadherin are expressed in only a small percentage of PC patients’ CTCs (Rhim et al., 2012). Previous studies have similarly demonstrated that (CK-/CD45-) circulating populations are heterogeneous and may consist not only of tumor cells that have undergone EMT (Zhang et al., 2015), but also of other tumor-associated cell populations, such as cCAFs, which have also been associated with adverse clinical outcomes. (Ao et al., 2015; Muchlińska et al., 2023). Therefore, although mesenchymal marker expression was not assessed in this cohort and the precise cellular composition of CTACs remains to be determined through molecular characterization, their enrichment in PC patients, strong discriminatory performance in ROC analysis, and association with adverse clinical outcomes support the potential biological relevance of these subpopulations and the importance of examining these (CK-/CD45-) cells in patients’ samples.

Regarding immune checkpoint molecules, PD-L1 and CTLA-4 are often overexpressed in CTCs and have been associated with patients’ clinical outcomes. This has been previously confirmed by our research group in TNBC, Non-small-cell lung cancer (NSCLC), and prostate cancer, highlighting that PD-L1 expression in CTCs is associated with less favorable patient survival and CTLA-4 expression correlates with metastatic disease (Vardas et al., 2023; Roumeliotou et al., 2024b). Most studies in PC have focused on immune checkpoint molecule expression in tumor tissues (Zhang et al., 2022; El-Anwar and El Nemr, 2021), whereas their direct assessment in CTCs remains unreported. Specifically, studies regarding PD-L1 expression in PC have yielded inconsistent results, with both comparatively high (Yamaki et al., 2017; Constantin et al., 2022) and low (Liang et al., 2018) expression levels reported in tissue studies and in meta-analyses (Gao et al., 2018). Similarly, reports of CTLA-4 expression in PC tumor cells are rare and generally at low levels (El-Anwar and El Nemr, 2021).

In the current study, PD-L1 and CTLA-4 expression in CTCs was low or absent (Figure 2D). However, patients with metastatic disease harboring PD-L1+ CTCs demonstrated worse OS (*p* = 0.025, Figure 2G.) Conversely, CTACs exhibited higher positivity for immune checkpoint molecules; whereas the phenotypes lacking PD-L1 and CTLA-4 expression remained the most prevalent (Figures 2E,F). Interestingly, PD-L1 expression on CTACs was observed more frequently in patients with metastatic disease compared with non-metastatic cases (Supplementary Table S4). Although the number of PD-L1-positive cases was limited and these findings should be considered exploratory, these observations are consistent with the low PD-L1 expression in tumor cells reported in PC tissue studies (Liang et al., 2018). They are also consistent with the higher frequency of PD-L1 positivity observed in more aggressive disease stages, such as metastatic settings, among different cancer types (Manjunath et al., 2019; Vardas et al., 2023). These preliminary findings support the notion of weak and inconsistent activation of PD-1/PD-L1 or CTLA-4 pathways, possibly reflecting the limited efficacy of immune checkpoint inhibitors in this disease (Yu et al., 2025; Minaei et al., 2024; Timmer et al., 2021).

The functional characteristics of SOCE components, STIM1 and ORAI1, have been reported to be upregulated in tissue samples and linked to enhanced proliferation (Khan et al., 2020), invasion (Okeke et al., 2016), and resistance to apoptosis (Kondratska et al., 2014) in PC. However, direct assessment of STIM1 and ORAI1 expression in CTCs has been reported only in prostate cancer patients, where their expression was linked to disease recurrence (Roumeliotou et al., 2024a).

In our study, STIM1 expression was observed in high frequency in CTACs, consistent with previous evidence in PDAC, where STIM1 knockdown reversed EMT, restoring epithelial features, further supporting a mechanistic link between STIM1 and this process (Wang et al., 2019). Moreover, the positive correlation observed between STIM1-positive and ORAI1-positive CTCs (Supplementary Table S11) indicates co-expression of these molecules in CTCs, consistent with our previous findings in prostate cancer (Roumeliotou et al., 2024a).

A correlation between STIM1 expression in CTCs and patient clinical outcomes was also identified. Specifically, the presence of STIM1-positive CTCs was associated with improved OS compared to patients without this phenotype (Figure 5A). Although this finding contrasts with our prior results in prostate cancer CTCs, where SOCE components were correlated with disease recurrence (Roumeliotou et al., 2024a), this paradoxical finding may reflect the context-dependent role of STIM1. Interestingly, publicly available datasets support that the relationship between STIM1 expression and disease progression is not uniform across malignancies. Analysis of the TNM plot database (https://tnmplot.com/analysis/) demonstrated significantly lower STIM1 expression in metastatic pancreatic tissue compared to primary tumor tissue. However, a statistically significant difference was observed, regarding OS, between high and low STIM1-expressing pancreatic primary tumors (Supplementary Figure S3). In contrast, Kaplan-Meier Plotter analysis (https://kmplot.com/analysis/) reveals an association between high STIM1 expression and improved overall survival in certain cancer types, including breast cancer, esophageal adenocarcinoma, kidney renal clear cell carcinoma, thymoma, and thyroid cancer (Supplementary Figure S3). Regarding the cellular mechanism, although in pancreatic tumor tissues, STIM1 has been shown to promote metastatic behavior under hypoxic conditions (Wang et al., 2019; Qi et al., 2016; Liu et al., 2015; Yang et al., 2018), excessive STIM1-mediated calcium entry could also trigger apoptosis through calcium overload under stress, as shown in gastric cancer cells treated with 3,3′-diindolylmethane (Ye et al., 2021). While CTCs are not directly exposed to such agents, they similarly circulate in a highly oxidative and mechanically stressful environment that influences their viability and signaling dynamics (Xin et al., 2020). These stress conditions might disturb CTC calcium homeostasis, with STIM1 expression increasing calcium influx beyond the cell’s threshold, triggering cell death. This could render STIM1-positive CTCs less capable of surviving during circulation or in the metastatic niche, possibly explaining the improved survival observed in PC patients. It is important to mention that this proposed mechanistic explanation, while biologically plausible and supported by previous studies, remains speculative and requires experimental validation, since these mechanisms were not directly addressed in this study.

The dual role of CD98hc, as part of heteromeric amino acid transporters and a β-integrin co-receptor, has been associated with malignant behavior in various cancer types (Park et al., 2024; Xia and Dubrovska, 2023). Elevated CD98hc expression in tumor tissue has been correlated with aggressiveness and poor clinical outcomes (Kaira et al., 2009; Ichinoe et al., 2021). However, emerging studies have reported variable CD98hc expression in PC tissues from low detection rates (13%–20%) (Bianconi et al., 2022) to higher expression levels (56.7%) (Kaira et al., 2012). While CD98hc expression was not identified as an independent prognostic factor in these studies, recent *in vitro* assays demonstrated that downregulation of CD98hc in PC cell lines significantly inhibited proliferation, self-renewal, and anchorage-independent growth, suggesting its involvement in maintaining malignant tumor features in PC (Bianconi et al., 2022).

Nevertheless, data regarding the expression of CD98hc at the CTC level were not previously documented. In our cohort, CD98hc-positive cancer cells were detected in 43% of PC patients (Figure 4C), consistent with previous tissue studies (Park et al., 2024; Kaira et al., 2009; Kaira et al., 2012). Particularly, Kaira et al. (2012) suggested that CD98hc expression was independently correlated with an unfavorable prognosis in patients with resectable adenocarcinoma and early-stage disease (Kaira et al., 2012). Similarly, in our cohort, the presence of ≥2 (CD98hc+/CD45-) cells was associated with significantly shorter PFS among patients who experienced disease progression within 12 months (Figure 5B). Notably, the precise contribution of CTCs and CTACs within the CD45-negative cell subpopulation remains to be determined because this study focused only on (CD98hc+/CD45-)cells, excluding CK antibody from the analysis, due to its limited expression in PC.

Although this study presents several limitations, including a limited cohort size, a low frequency of CK-positive CTCs, and the absence of molecular validation, the results highlight the inter- and intra-heterogeneity of CTCs in PC patients and emphasize the potential biological relevance of CTAC subpopulations. The assessment of immune checkpoint molecules and SOCE pathway molecules expression revealed phenotypic patterns that further confirm the unique biology of PC, including its limited response to immunotherapy and its aggressive clinical outlook. Furthermore, the association between clinical outcomes and the expression of PD-L1, STIM1, and CD98hc suggests these biomarkers possess circulating biological relevance. However, these findings remain exploratory (hypothesis-generating) due to the small patient number, and multivariable analyses did not consistently support their independent clinical value. Further studies are needed to molecularly validate the malignant nature of CTACs, through the assessment of genetic instability and EMT markers, as well as through a larger patient cohort and functional confirmation of the proposed mechanisms. In addition, a larger cohort of healthy donors would be necessary to confirm the threshold in ROC analysis. This approach will allow a better understanding of the phenotypic profiles of malignant cells in PC patients and may uncover novel, clinically meaningful targets for disease monitoring and therapeutic intervention.

To the best of our knowledge, this is the first study to assess the above-mentioned combination of biomarkers, including immune checkpoint and calcium signaling molecules in morphologically and phenotypically defined tumor-derived cells from PC patients, exploring their potential prognostic significance. CTACs emerged as the predominant subpopulation, emphasizing the limitations of conventional epithelial marker-based CTC detection. ROC analysis confirmed their strong ability to discriminate between PC patients and healthy donors. Key biomarkers, including PD-L1, STIM1, and ORAI1 and CD98hc were detected in circulating cells of tumor origin, with PD-L1, CD98hc and STIM1 significantly associated with differential clinical outcomes. Overall, the observed expression patterns suggest mechanisms linked to immune checkpoint inhibitor resistance and implicate calcium signaling and amino acid transport pathways in CTC-mediated disease progression.

## Data Availability

The original contributions presented in the study are included in the article/Supplementary Material, further inquiries can be directed to the corresponding author.
