# Supplementary material for: The phenotypic heterogeneity of tumor-derived cells in the bloodstream of pancreatic cancer patients
Source: Front Cell Dev Biol. 2026 Sep 14;14:1881239. doi: 10.3389/fcell.2026.1881239 (PMC13617025; doi:10.3389/fcell.2026.1881239)
Supplement: Supplementary file 1 [file DataSheet1.docx]

Supplementary Material

# 1 Supplementary Tables

## 1.1 Supplementary table S1. (CD45-) cells detected in healthy donor (HD) peripheral blood mononuclear cells (PBMCs) in staining (STIM1/CD45).

| HD No. | Total (CD45-) cells | (STIM1+/CD45-) | (STIM1-/CD45-) |
| --- | --- | --- | --- |
| 1 | 0 | 0 | 0 |
| 2 | 4 | 0 | 4 |
| 3 | 3 | 1 | 0 |
| 4 | 1 | 1 | 0 |
| 5 | 0 | 0 | 0 |
| 6 | 1 | 1 | 0 |
| 7 | 2 | 1 | 1 |
| 8 | 2 | 0 | 0 |
| 9 | 1 | 1 | 0 |
| 10 | 0 | 0 | 0 |

## 1.2 Supplementary table S2. (Circulating tumor cells (CTCs) and circulating tumor-associated cells (CTACs) detected in pancreatic cancer (PC) patient using size-based ISET filtration.

| Patient No. | Total CTCs | Total CTACs |
| --- | --- | --- |
| 1 | 0 | 5 |
| 2 | 4 | 65 |
| 3 | 0 | 4 |
| 4 | 1 | 6 |
| 5 | 0 | 8 |
| 6 | 0 | 25 |
| 7 | 0 | 4 |
| 8 | 2 | 17 |
| 9 | 0 | 0 |
| 10 | 0 | 5 |
| 11 | 2 | 102 |
| 12 | 0 | 32 |
| 13 | 0 | 27 |
| 14 | 0 | 12 |
| 15 | 0 | 2 |

## 1.3 Supplementary table S3. Circulating tumor cells (CTCs) and circulating tumor-associated cells (CTACs) detected in pancreatic cancer (PC) patients in staining (CK/PD-L1/CD45).

| Patient No. | Total CTCs | (CK+/PD-L1+) | (CK+/PD-L1-) | Total CTACs | (CK-/PD-L1+) | (CK-/PD-L1-) |
| --- | --- | --- | --- | --- | --- | --- |
| 1 | 1 | 1 | 0 | 1 | 0 | 1 |
| 2 | 0 | 0 | 0 | 6 | 1 | 5 |
| 3 | 0 | 0 | 0 | 1 | 0 | 1 |
| 4 | 0 | 0 | 0 | 1 | 0 | 1 |
| 5 | 0 | 0 | 0 | 4 | 0 | 4 |
| 6 | 1 | 0 | 1 | 4 | 0 | 4 |
| 7 | 0 | 0 | 0 | 8 | 1 | 7 |
| 8 | 0 | 0 | 0 | 0 | 0 | 0 |
| 9 | 0 | 0 | 0 | 4 | 1 | 3 |
| 10 | 0 | 0 | 0 | 1 | 0 | 1 |
| 11 | 0 | 0 | 0 | 1 | 0 | 1 |
| 12 | 0 | 0 | 0 | 0 | 0 | 0 |
| 13 | 0 | 0 | 0 | 0 | 0 | 0 |
| 14 | 0 | 0 | 0 | 1 | 0 | 1 |
| 15 | 0 | 0 | 0 | 5 | 3 | 2 |
| 16 | 0 | 0 | 0 | 8 | 3 | 5 |
| 17 | 0 | 0 | 0 | 1 | 1 | 0 |
| 18 | 0 | 0 | 0 | 15 | 1 | 14 |
| 19 | 0 | 0 | 0 | 11 | 0 | 11 |
| 20 | 0 | 0 | 0 | 2 | 0 | 2 |
| 21 | 0 | 0 | 0 | 2 | 1 | 1 |
| 22 | 0 | 0 | 0 | 1 | 0 | 1 |
| 23 | 0 | 0 | 0 | 0 | 0 | 0 |
| 24 | 1 | 0 | 1 | 7 | 2 | 5 |
| 25 | 0 | 0 | 0 | 3 | 0 | 3 |
| 26 | 0 | 0 | 0 | 3 | 2 | 1 |
| 27 | 0 | 0 | 0 | 1 | 0 | 1 |
| 28 | 0 | 0 | 0 | 0 | 0 | 0 |
| 29 | 0 | 0 | 0 | 1 | 0 | 1 |
| 30 | 0 | 0 | 0 | 2 | 1 | 1 |
| 31 | 0 | 0 | 0 | 1 | 0 | 1 |
| 32 | 0 | 0 | 0 | 3 | 0 | 3 |
| 33 | 0 | 0 | 0 | 2 | 1 | 1 |
| 34 | 0 | 0 | 0 | 6 | 0 | 6 |
| 35 | 0 | 0 | 0 | 0 | 0 | 0 |
| 36 | 0 | 0 | 0 | 0 | 0 | 0 |
| 37 | 0 | 0 | 0 | 13 | 0 | 13 |
| 38 | 0 | 0 | 0 | 2 | 0 | 2 |
| 39 | 0 | 0 | 0 | 0 | 0 | 0 |
| 40 | 0 | 0 | 0 | 2 | 0 | 2 |

## 1.4  **Supplementary table S4. Circulating tumor cells (CTCs) and circulating tumor-associated cells (CTACs) detected in non-metastatic and metastatic pancreatic cancer (PC) patients in staining (CK/PD-L1/CD45).**

**A)**

| **Patients’ Phenotypes** | **CTC+ patients** | | **CTAC+ patients** | |
| --- | --- | --- | --- | --- |
|  | PD-L1+ CTCs | PD-L1- CTCs | PD-L1+ CTACs | PD-L1- CTACs |
| **Non-Metastatic** | 50 (1/2) | 50 (1/2) | 20 (2/10) | 90 (9/10) |
| **Metastatic** | 0 | 100 (1/1) | 55 (6/11) | 82 (9/11) |

**B)**

| **Cells phenotypes** | **CTCs** | | **CTACs** | |
| --- | --- | --- | --- | --- |
|  | PD-L1+ | PD-L1- | PD-L1+ | PD-L1- |
| **Non-metastatic** | 50 (1/2) | 50 (1/2) | 11 (4/37) | 89 (33/37) |
| **Metastatic** | 0 | 100 (1/1) | 14 (9/63) | 86 (54/63) |

## 1.5 Supplementary table S5. Circulating tumor cells (CTCs) and circulating tumor-associated cells (CTACs) detected in pancreatic cancer (PC) patients in staining (CK/CTLA-4/CD45).

| Patient No. | Total CTCs | (CK+/CTLA-4+) | (CK+/CTLA-4-) | Total CTACs | (CK-/CTLA-4+) | (CK-/CTLA-4-) |
| --- | --- | --- | --- | --- | --- | --- |
| 1 | 0 | 0 | 0 | 8 | 0 | 8 |
| 2 | 0 | 0 | 0 | 1 | 0 | 1 |
| 3 | 0 | 0 | 0 | 1 | 0 | 1 |
| 4 | 0 | 0 | 0 | 0 | 0 | 0 |
| 5 | 0 | 0 | 0 | 7 | 0 | 7 |
| 6 | 0 | 0 | 0 | 14 | 0 | 14 |
| 7 | 0 | 0 | 0 | 8 | 0 | 8 |
| 8 | 0 | 0 | 0 | 2 | 2 | 0 |
| 9 | 0 | 0 | 0 | 5 | 0 | 5 |
| 10 | 0 | 0 | 0 | 2 | 0 | 2 |
| 11 | 0 | 0 | 0 | 1 | 0 | 1 |
| 12 | 0 | 0 | 0 | 1 | 0 | 1 |
| 13 | 0 | 0 | 0 | 0 | 0 | 0 |
| 14 | 0 | 0 | 0 | 0 | 0 | 0 |
| 15 | 0 | 0 | 0 | 6 | 1 | 5 |
| 16 | 0 | 0 | 0 | 6 | 0 | 6 |
| 17 | 0 | 0 | 0 | 7 | 2 | 5 |
| 18 | 0 | 0 | 0 | 3 | 0 | 3 |
| 19 | 0 | 0 | 0 | 13 | 0 | 13 |
| 20 | 0 | 0 | 0 | 11 | 0 | 11 |
| 21 | 0 | 0 | 0 | 5 | 0 | 5 |
| 22 | 0 | 0 | 0 | 3 | 0 | 3 |
| 23 | 0 | 0 | 0 | 4 | 0 | 4 |
| 24 | 0 | 0 | 0 | 4 | 0 | 4 |
| 25 | 0 | 0 | 0 | 4 | 1 | 3 |
| 26 | 0 | 0 | 0 | 0 | 0 | 0 |
| 27 | 0 | 0 | 0 | 0 | 0 | 0 |
| 28 | 0 | 0 | 0 | 1 | 0 | 1 |
| 29 | 0 | 0 | 0 | 2 | 1 | 1 |
| 30 | 0 | 0 | 0 | 3 | 0 | 3 |
| 31 | 0 | 0 | 0 | 2 | 0 | 2 |
| 32 | 0 | 0 | 0 | 0 | 0 | 0 |
| 33 | 0 | 0 | 0 | 1 | 1 | 0 |
| 34 | 0 | 0 | 0 | 5 | 0 | 5 |
| 35 | 0 | 0 | 0 | 11 | 1 | 10 |
| 36 | 0 | 0 | 0 | 3 | 0 | 3 |
| 37 | 0 | 0 | 0 | 6 | 0 | 6 |
| 38 | 0 | 0 | 0 | 6 | 0 | 6 |
| 39 | 0 | 0 | 0 | 1 | 0 | 1 |
| 40 | 0 | 0 | 0 | 1 | 0 | 1 |

## 1.6 Supplementary table S6. Circulating tumor cells (CTCs) and circulating tumor-associated cells (CTACs) detected in non-metastatic and metastatic pancreatic cancer (PC) patients in staining (CK/CTLA-4/CD45).

**A)**

| **Patients’ Phenotypes** | **CTC+ patients** | | **CTAC+ patients** | |
| --- | --- | --- | --- | --- |
|  | CTLA-4+ CTCs | CTLA-4- CTCs | CTLA-4+ CTACs | CTLA-4 - CTACs |
| **Non-Metastatic** | 0 | 0 | 20 (2/10) | 60 (6/10) |
| **Metastatic** | 0 | 0 | 18 (2/11) | 100 (11/11) |

**B)**

| **Cells phenotypes** | **CTCs** | | **CTACs** | |
| --- | --- | --- | --- | --- |
|  | CTLA-4+ | CTLA-4- | CTLA-4 + | CTLA-4 - |
| **Non-metastatic** | 0 | 0 | 4 (2/49) | 96 (47/49) |
| **Metastatic** | 0 | 0 | 3 (2/70) | 97 (68/70) |

## 1.7 Supplementary table S7. Circulating tumor cells (CTCs) and circulating tumor-associated cells (CTACs) detected in pancreatic cancer (PC) patients in staining (CK/STIM1/CD45).

| Patient No. | Total CTCs | (CK+/STIM1+) | (CK+/STIM1-) | Total CTACs | (CK-/STIM1+) | (CK-/STIM1-) |
| --- | --- | --- | --- | --- | --- | --- |
| 1 | 1 | 1 | 0 | 8 | 0 | 8 |
| 2 | 0 | 0 | 0 | 5 | 1 | 4 |
| 3 | 0 | 0 | 0 | 13 | 2 | 11 |
| 4 | 0 | 0 | 0 | 13 | 0 | 13 |
| 5 | 0 | 0 | 0 | 13 | 3 | 10 |
| 6 | 0 | 0 | 0 | 9 | 0 | 9 |
| 7 | 0 | 0 | 0 | 6 | 0 | 6 |
| 8 | 1 | 1 | 0 | 27 | 2 | 25 |
| 9 | 1 | 1 | 0 | 31 | 10 | 21 |
| 10 | 0 | 0 | 0 | 8 | 1 | 7 |
| 11 | 0 | 0 | 0 | 9 | 2 | 7 |
| 12 | 0 | 0 | 0 | 7 | 1 | 6 |
| 13 | 0 | 0 | 0 | 6 | 3 | 3 |
| 14 | 0 | 0 | 0 | 9 | 1 | 8 |
| 15 | 0 | 0 | 0 | 10 | 2 | 8 |
| 16 | 0 | 0 | 0 | 4 | 1 | 3 |
| 17 | 0 | 0 | 0 | 4 | 4 | 0 |
| 18 | 0 | 0 | 0 | 11 | 5 | 6 |
| 19 | 0 | 0 | 0 | 11 | 1 | 10 |
| 20 | 0 | 0 | 0 | 10 | 3 | 7 |
| 21 | 0 | 0 | 0 | 4 | 4 | 0 |
| 22 | 0 | 0 | 0 | 6 | 4 | 2 |
| 23 | 2 | 1 | 1 | 41 | 28 | 13 |
| 24 | 0 | 0 | 0 | 15 | 4 | 11 |
| 25 | 0 | 0 | 0 | 3 | 2 | 1 |
| 26 | 0 | 0 | 0 | 8 | 1 | 7 |
| 27 | 0 | 0 | 0 | 4 | 0 | 4 |
| 28 | 0 | 0 | 0 | 3 | 0 | 3 |
| 29 | 0 | 0 | 0 | 6 | 1 | 5 |
| 30 | 0 | 0 | 0 | 5 | 0 | 5 |
| 31 | 0 | 0 | 0 | 2 | 0 | 2 |
| 32 | 0 | 0 | 0 | 9 | 0 | 9 |
| 33 | 0 | 0 | 0 | 7 | 2 | 5 |
| 34 | 0 | 0 | 0 | 24 | 2 | 22 |
| 35 | 0 | 0 | 0 | 5 | 4 | 1 |
| 36 | 0 | 0 | 0 | 3 | 0 | 3 |
| 37 | 1 | 1 | 0 | 45 | 6 | 39 |
| 38 | 2 | 1 | 1 | 0 | 0 | 0 |
| 39 | 0 | 0 | 0 | 4 | 1 | 3 |
| 40 | 0 | 0 | 0 | 0 | 0 | 0 |

## 1.8 Supplementary table S8. Circulating tumor cells (CTCs) and circulating tumor-associated cells (CTACs) detected in non-metastatic and metastatic pancreatic cancer (PC) patients in staining (CK/STIM1/CD45).

**A)**

| **Patients’ Phenotypes** | **CTC+ patients** | | **CTAC+ patients** | |
| --- | --- | --- | --- | --- |
|  | STIM1+ CTCs | STIM1- CTCs | STIM1+ CTACs | STIM1- CTACs |
| **Non-Metastatic** | 100 (5/5) | 20 (1/5) | 90 (9/10) | 100 (10/10) |
| **Metastatic** | 100 (1/1) | 100 (1/1) | 91 (10/11) | 91 (10/11) |

**B)**

| **Cells phenotypes** | **CTCs** | | **CTACs** | |
| --- | --- | --- | --- | --- |
|  | STIM1+ | STIM1- | STIM1+ | STIM1- |
| **Non-metastatic** | 83 (5/6) | 17 (1/6) | 14 (24/167) | 86 (143/167) |
| **Metastatic** | 50 (1/2) | 50 (1/2) | 28 (30/107) | 72 (77/107) |

## 1.9 Supplementary table S9. Circulating tumor cells (CTCs) and circulating tumor-associated cells (CTACs) detected in pancreatic cancer (PC) patients in staining (CK/ORAI1/CD45).

| Patient No. | Total CTCs | (CK+/ORAI1+) | (CK+/ORAI1-) | Total CTACs | (CK-/ORAI1+) | (CK-/ORAI1-) |
| --- | --- | --- | --- | --- | --- | --- |
| 1 | 2 | 2 | 0 | 12 | 1 | 11 |
| 2 | 0 | 0 | 0 | 7 | 0 | 7 |
| 3 | 0 | 0 | 0 | 8 | 0 | 8 |
| 4 | 0 | 0 | 0 | 6 | 0 | 6 |
| 5 | 0 | 0 | 0 | 5 | 0 | 5 |
| 6 | 0 | 0 | 0 | 16 | 0 | 16 |
| 7 | 0 | 0 | 0 | 6 | 0 | 6 |
| 8 | 0 | 0 | 0 | 2 | 0 | 2 |
| 9 | 0 | 0 | 0 | 13 | 0 | 13 |
| 10 | 0 | 0 | 0 | 0 | 0 | 0 |
| 11 | 0 | 0 | 0 | 1 | 1 | 0 |
| 12 | 0 | 0 | 0 | 2 | 0 | 2 |
| 13 | 0 | 0 | 0 | 0 | 0 | 0 |
| 14 | 0 | 0 | 0 | 0 | 0 | 0 |
| 15 | 0 | 0 | 0 | 1 | 0 | 1 |
| 16 | 0 | 0 | 0 | 1 | 0 | 1 |
| 17 | 0 | 0 | 0 | 3 | 0 | 3 |
| 18 | 0 | 0 | 0 | 24 | 0 | 24 |
| 19 | 0 | 0 | 0 | 18 | 0 | 18 |
| 20 | 0 | 0 | 0 | 6 | 0 | 6 |
| 21 | 0 | 0 | 0 | 8 | 0 | 8 |
| 22 | 0 | 0 | 0 | 15 | 0 | 15 |
| 23 | 0 | 0 | 0 | 121 | 7 | 114 |
| 24 | 0 | 0 | 0 | 65 | 0 | 65 |
| 25 | 0 | 0 | 0 | 62 | 0 | 62 |
| 26 | 0 | 0 | 0 | 0 | 0 | 0 |
| 27 | 0 | 0 | 0 | 17 | 0 | 17 |
| 28 | 0 | 0 | 0 | 0 | 0 | 0 |
| 29 | 0 | 0 | 0 | 2 | 0 | 2 |
| 30 | 0 | 0 | 0 | 0 | 0 | 0 |
| 31 | 0 | 0 | 0 | 10 | 0 | 10 |
| 32 | 0 | 0 | 0 | 0 | 0 | 0 |
| 33 | 0 | 0 | 0 | 3 | 0 | 3 |
| 34 | 0 | 0 | 0 | 13 | 0 | 13 |
| 35 | 0 | 0 | 0 | 5 | 1 | 4 |
| 36 | 0 | 0 | 0 | 1 | 0 | 1 |
| 37 | 0 | 0 | 0 | 8 | 0 | 8 |
| 38 | 0 | 0 | 0 | 2 | 0 | 2 |
| 39 | 0 | 0 | 0 | 3 | 0 | 3 |
| 40 | 0 | 0 | 0 | 6 | 0 | 6 |

## 1.10 Supplementary table S10. Circulating tumor cells (CTCs) and circulating tumor-associated cells (CTACs) detected in non-metastatic and metastatic pancreatic cancer (PC) patients in staining (CK/ORAI1/CD45).

**A)**

| **Patients’ Phenotypes** | **CTC+ patients** | | **CTAC+ patients** | |
| --- | --- | --- | --- | --- |
|  | ORAI1+ CTCs | ORAI1- CTCs | ORAI1 + CTACs | ORAI1 - CTACs |
| **Non-Metastatic** | 100 (1/1) | 0 | 10 (1/10) | 100 (10/10) |
| **Metastatic** | 0 | 0 | 9 (1/11) | 100 (11/11) |

**B)**

| **Cells phenotypes** | **CTCs** | | **CTACs** | |
| --- | --- | --- | --- | --- |
|  | ORAI1+ | ORAI1- | ORAI1 + | ORAI1 - |
| **Non-metastatic** | 100 (2/2) | 0 | 1 (1/88) | 99 (87/88) |
| **Metastatic** | 0 | 0 | 1(1/168) | 99 (167/168) |

## 1.11 Supplementary table S11. Spearman correlations between STIM1-positive circulating tumor cells (CTCs) and ORAI1 expression phenotypes.

|  | **rho** | ***p*-value** |
| --- | --- | --- |
| STIM1+ CTCs and ORAI1+ CTCs | 0.348 | 0.028 |
| STIM1+ CTCs and ORAI1+ CTACs | 0.504 | <0.001 |

## 1.12 Supplementary table S12. (CD45-) cells detected in pancreatic cancer (PC) patients in staining (CD98hc/CD45).

| Patient No. | Total (CD45-) cells | (CD98hc+/CD45-) | (CD98hc-/CD45-) |
| --- | --- | --- | --- |
| 1 | 2 | 1 | 1 |
| 2 | 9 | 1 | 8 |
| 3 | 5 | 0 | 5 |
| 4 | 5 | 1 | 4 |
| 5 | 4 | 0 | 4 |
| 6 | 8 | 0 | 8 |
| 7 | 8 | 3 | 5 |
| 8 | 12 | 1 | 11 |
| 9 | 8 | 0 | 8 |
| 10 | 4 | 0 | 4 |
| 11 | 2 | 1 | 1 |
| 12 | 6 | 0 | 6 |
| 13 | 9 | 0 | 9 |
| 14 | 0 | 0 | 0 |
| 15 | 6 | 0 | 6 |
| 16 | 7 | 3 | 4 |
| 17 | 9 | 0 | 9 |
| 18 | 14 | 3 | 11 |
| 19 | 3 | 0 | 3 |
| 20 | 5 | 0 | 5 |
| 21 | 2 | 0 | 2 |
| 22 | 5 | 0 | 5 |
| 23 | 11 | 1 | 10 |
| 24 | 11 | 0 | 11 |
| 25 | 27 | 2 | 25 |
| 26 | 0 | 0 | 0 |
| 27 | 6 | 0 | 6 |
| 28 | 0 | 0 | 0 |
| 29 | 0 | 0 | 0 |
| 30 | 0 | 0 | 0 |
| 31 | 0 | 0 | 0 |
| 32 | 0 | 0 | 0 |
| 33 | 0 | 0 | 0 |
| 34 | 4 | 0 | 4 |
| 35 | 1 | 1 | 0 |
| 36 | 0 | 0 | 0 |
| 37 | 14 | 5 | 9 |
| 38 | 0 | 0 | 0 |
| 39 | 3 | 3 | 0 |
| 40 | 4 | 0 | 4 |

## 1.13 Supplementary table S13. Circulating tumor cells (CTCs) and circulating tumor-associated cells (CTACs) detected in non-metastatic and metastatic pancreatic cancer (PC) patients in staining (CD98hc/CD45).

**A)**

| **Patients' phenotypes** | **(CD45-) cells** | |
| --- | --- | --- |
|  | CD98hc+ | CD98hc- |
| **Non-metastatic** | 50 (6/12) | 92 (11/12) |
| **Metastatic** | 35 (6/17) | 94 (16/17) |

**B)**

| **Cells phenotypes** | **(CD45-) cells** | |
| --- | --- | --- |
|  | CD98hc+ | CD98hc- |
| **Non-metastatic** | 17 (6/83) | 83 (69/83) |
| **Metastatic** | 10 (10/104) | 90 (94/104) |

# 2 Supplementary Figures

**Supplementary figure 1.** Expected marker expression in MIA PaCa-2 cells spiked into healthy donor peripheral blood mononuclear cells (PBMCs) across stainings. (A) (CK/PD-L1/CD45) staining, stained with CK (red), PD-L1 (green) and CD45 (purple). (B) (CK/CTLA-4/CD45) staining, stained with CK (red), CTLA-4 (green) and CD45 (purple). (C) (CK/STIM1/CD45) staining, stained with CK (green), STIM1 (red), and CD45 (purple). (D) (CK/ORAI1/CD45) staining, stained with CK (red), ORAI1 (purple), and CD45 (green). Nuclei were stained with DAPI (blue). Images were acquired using the VyCAP platform at 20× magnification. Scale bars represent 10 μm.

**Supplementary figure 2.** Expression of CD98hc (purple), CD45 (green) in MDA-MB-231 cells spiked into healthy donor peripheral blood mononuclear cells (PBMCs). Nuclei were stained with DAPI (blue). Images were acquired using the VyCAP platform at 20× magnification. Scale bars represent 10 μm.

**Supplementary figure 3.** STIM1 expression and disease progression across cancer types. (A) TNM violin plot of STIM1 gene expression in normal, tumor and metastatic pancreatic tissue. Violin plots showing data density and mean/standard deviation for each tissue type. (B) Kaplan-Meier Plotter analysis of overall survival (OS) according to STIM1 expression in pancreatic cancer patients showing minimal differences between high and low STIM1-expressing tumors (C-G) Kaplan-Meier Plotter analyses of OS according to STIM1 expression in breast, thymoma, esophageal, thyroid, and kidney cancer patients, respectively, demonstrating a trend toward improved OS among patients with high STIM1-expressing tumors.
